# Understanding organizational and cultural premises for quality of care in nursing homes: an ethnographic study

**DOI:** 10.1186/s12913-015-1171-y

**Published:** 2015-11-13

**Authors:** Sigrid Nakrem

**Affiliations:** Faculty of Nursing, Sør-Trøndelag University College, P.O. Box 2004, 7004 Trondheim, Norway

**Keywords:** Ethnography, Nursing home, Organizational culture, Qualitative method, Quality improvement, Resident, Staff

## Abstract

**Background:**

Internationally, there are concerns about the quality of care in nursing homes. The concept of ‘corporate culture’ as an internal variable could be seen as the means to improve quality of care and quality of life for the residents. The aim of this article was to describe the nursing home culture from the staff’s perspective and to include how the residents describe quality of care.

**Methods:**

An ethnographic design was employed. A purposive sample of four municipal public nursing homes in Norway with long-term care residents was included in the study. Data were collected by participant observation including informal conversation with the staff, and in-depth interviews with 15 residents using a narrative approach.

**Results:**

The main findings were that organizational cultures could be seen as relatively stable corporate cultures described as ‘personalities’ with characteristics that were common for all nursing homes (conformity) and typical traits that were present in some nursing homes, but that they were also like no other nursing home (distinctiveness). Conformity (‘Every nursing home is like all other nursing homes’) meant that nursing home organizations formed their services according to a perception of what residents in general need and expect. Trait (‘Every nursing home is like some other nursing homes’) expressed typologies of nursing homes: residency, medical, safeguard or family orientation. The distinctness of each nursing home (‘Every nursing home is like no other nursing home’) was expressed in unique features of the nursing home; the characteristics of the nursing home involved certain patterns of structure, cultural assumptions and interactions that were unique in each nursing home. Nursing home residents experienced quality of care as ‘The nursing home as my home’ and ‘Interpersonal care quality’. The resident group in the different types of nursing homes were unique, and the experience of quality of care seemed to depend on whether their unique needs and expectations were met or not.

**Conclusion:**

In order to create a sustainable nursing home service the service needs to be characterized by learning and openness to change and must actually implement practices that respond to the resident and his or her family’s values.

**Electronic supplementary material:**

The online version of this article (doi:10.1186/s12913-015-1171-y) contains supplementary material, which is available to authorized users.

## Background

With the shifting demographic towards an ageing population in Western societies, nursing homes will continue to be an essential service provided to individuals for the foreseeable future. In Norway and many other European countries, elder care is recognized as a public responsibility. Norwegian municipalities provide long-term care in nursing homes to more than 41 000 people, i.e. one-fifth of the population over 80 years old [1]. Most nursing home residents have advanced chronic illnesses and multiple diagnoses with as many as 80 % suffering from dementia [2]. For long-term residents, the nursing home provides a complete service, including advanced health care, housing and social care [3]. End-of-life care is increasingly the responsibility of nursing homes, and over 45 % of all deaths occur in nursing homes [1]. The many functions of the nursing home and the diversity of the residents’ needs, varying from palliative care to social stimulation, add complexity to nursing care [4]. Therefore, to develop high-quality nursing home services that are suited for the future, nursing home organizations must adapt to these complexities.

According to Donabedian (1980), quality of care can be divided into at least two interrelating aspects: technical care, defined as the application of the science and technology of health science to the management of health problems; and interpersonal processes, specifically, the psychosocial interaction between client and practitioner. Technical care quality can be defined as the extent to which the care provided maximizes the health benefits without increasing risk, a valuation that must be shared by the patient and the practitioner [5]. Three quality domains should be considered when judging total quality: structure quality, comprising quality of the structural factors that affect the performance of care; process quality, or the quality of the direct care that the staff provides; and outcome quality, encompassing the impact for the patient or health care service outcome for the population. A variety of factors affect the processes and structure quality, which again indirectly provide the results for the individual patient, or the outcome of the service offered [6].

Quality in the interpersonal domain is measured by the degree of adherence to socially accepted values, which are reinforced by the ethical principles of health professions, and expectations of individual patients [5]. Client - nurse interaction is a major aspect in nursing [7], and variables related to client-nurse interactions include: the actors (client and nurse), social context for contact, process of interaction, and client health outcomes [7, 8]. Residents in nursing homes develop long-term relationships with nurses that require a unique approach to the interpersonal aspects of nursing. It has been found that the nurse-patient interaction is a vital resource for promoting physical, emotional, functional, social and spiritual well-being among nursing home patients [9]. In nursing homes the nursing care should take a holistic view [10–12], and person-centred care means to adopt the resident’s perspective resulting in a recognition of the resident’s and the family’s values [13]. High quality in dementia care means to help the resident to maintain a sense of personal worth, an ability to control his or her personal life, social confidence and hope in a situation where his or her dependence on others is prominent and increasing [14, 15].

The interpersonal relationship is regarded as an essential factor in person-centred care and interpersonal skills are considered part of nurses’ professional competence and a prerequisite for person-centred processes resulting in high quality of care [16, 17]. Hobbs (2009) conducted a dimensional analysis of the concept ‘patient-centred care’, and the central organizing perspective was that care quality is strongly connected to patient-nurse interaction, and nurses’ competences to alleviate the patient’s vulnerabilities [18].

Patient-centredness is a highly appreciated value stated in laws and regulations internationally. For instance, the Norwegian regulation for quality of nursing care in health and social services [19] states that the regulation has as its purpose to ‘assure that users of health and social services have their basic needs met, acknowledging the individual’s right to self-determination, value of selfhood and individual life style’ (p1). Laws regulating patients’ rights also underpin the right to participate in decisions regarding their own health as a central principle [20]. Another example is the National Minimum Standards for Care Homes for Older People (UK, England) which has a section related to shared decision-making, choice and control over one’s own life (Standard 14-Autonomy and Choice) [21, 22].

Most studies of nursing homes have dealt with the quality of medical care and the clinical conditions of the residents [23–25]. However, nursing homes have many additional functions for the long-term resident including as a home, the main social environment and a complete health care service. Since the nursing home could be understood as a community for those who live, visit and work there [26], it might be useful to study the nursing home’s organizational culture. Researchers have held varying conceptions of culture, and they have drawn from both organization theory and social anthropology [27]. In addition, there is little agreement among scholars as to what the terms organization and culture mean, and how each can be observed or measured [28].

Organizational culture is defined as a set of values, beliefs, norms, customs, rules, and codes that lead people to define themselves as a distinct group with a sense of commonality [29]. Whereas ‘corporate culture’ is defined as a value-infused institution, complete with artefacts, symbolic codes of behaviour, rituals and specialized language commonly held by all it employees [28]. The corporate culture has as a set of social practices within the organization that brings people together. Norms and values are learned as part of the cultural conditioning, and they shape the way people view the world and how they interact with one another [28]. Communication and interaction reinforces the process, and the culture is regarded as the social and normative ‘glue’ that holds an organization together [30]. Although organizations, such as nursing homes, are embedded within a wider cultural context, they are also culture-producing phenomena [27]. The corporate culture affects each employee in the nursing home and, in turn, the employee takes an active part in re-creating the corporate culture through networking with other employees. Nursing homes are often seen as having strong corporate cultures with limited interaction with the communal society outside the organization [31, 32]. Nursing home residents are often perceived as passive receivers of care, thus, mainly employees and the organization itself are presumed to produce the culture [28].

Organizational culture can be understood as either something that an organization has, more specific a corporate culture as an internal variable, or something an organization is, conceptualizing culture as a root metaphor [28]. Culture as an internal variable builds on the assumption that corporate cultures are dynamic and evolving. Culture is understood as an internal organizational variable that can be shaped in particular ways to change or improve the organization [27, 28]. In the present study, the focus is on development of nursing home organizations. Quality improvements in the nursing homes’ culture could be seen as a means to achieve quality of care and quality of life for nursing home residents [33]. Interventions are often directed at the organization’s corporate culture and aim at questioning the espoused values and underlying assumptions under which employees operate [28]. According to Smircich (1983), the conception of an organization as a culture includes an examination of symbolic aspects of social practices within the organization. Therefore, to study the culture, symbolic artefacts and codes by which the participants themselves make sense of their experience and how this relates to their behaviours are observed and interpreted. This study aimed to describe the nursing homes as corporate cultures from the staff’s perspective, and includes how cognitively competent residents describe quality of care. An additional aim was to acquire a better understanding of this link in order to create better nursing homes for the future.

## Methods

This study is part of a larger study aiming at exploring the most important dimensions of quality of care in nursing homes by describing the perspectives of residents, family and staff [34]. In the present study, two data collection approaches were employed: 1) an ethnographic design using participating observation; and 2) in-depth interviews with residents. A systematic approach to everyday life in the natural setting of nursing homes was used to illuminate the specific research questions, carefully interpreted to draw valid meaning from these data. The purpose was to describe what happens, how the people involved see and talk about their own actions and those of others, the contexts in which the action takes place, and what follows from it [35]. The findings from resident and family interviews have been published previously [4, 36, 37]. However, in the present article, materials from resident interviews have been used in view of the findings achieved from field notes. The reason for this new approach was that the resident interviews represented only cognitively competent residents, whereas the observations included all residents, both cognitively competent and cognitively impaired, as well as the nursing homes’ organization and corporate cultures.

### Setting and study participants

A purposive sample [38] of four municipal public nursing homes in Norway with long-term care residents was included. Research indicates that there are differences between small-, medium- and large-sized nursing homes and in urban and rural areas [39, 40]. Therefore, a sample of nursing homes that reflects these features was selected. For the purpose of this study, the four nursing homes were given pseudonyms, namely Residence, Hospital, Shelter and Village. The four nursing homes had mixed populations in regard to medical diagnoses, physical and cognitive functioning, ages and gender.

### Data collection

**Fig. 1:**
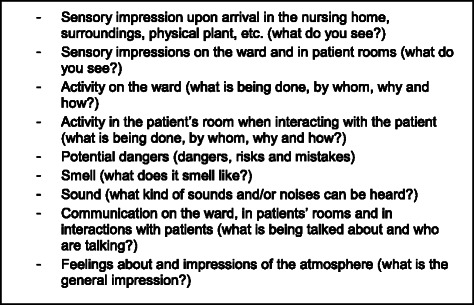
Observational guide

The data were collected in 2008 by participant observation, informal conversations and discussions with staff, document studies in the four nursing homes, and in addition in-depth interviews with the residents. The researcher first contacted the management of each nursing home and received permission to do the study. The staff, residents and relatives were informed about the study and information pamphlets were distributed. Information notices were placed on front doors, notice boards and in a ring binder in each nursing home’s staff room. There were opportunities for asking for clarification at all times when the observers were present, or anyone could ask to see the ring binder for more information. The author of this paper (researcher) and a research assistant entered the units, wearing health workers’ clothing, and participated in daily activities related to nursing care and practical tasks in the nursing home. Both observers are registered nurses (RNs) and postgraduate specialists. The two observers were present simultaneously at each of the nursing homes for 4 or 5 days and, on average for 5 h per day in the morning or afternoon/evening. The total observation time was 195 h with 44 to 52 h spent in each nursing home. An observational guide was used by both observers, see Fig. 1. Consent from the residents and staff members was continuously collected orally by asking permission to observe and assuring that the resident being observed did not object to the observation. If there were any indications such as signs of discomfort among the residents or negative statements from the resident or a family member that were perceived as doubt of consent, the observers left the room and no notes were taken. Observations focused on organizational structure, practical tasks and activities on the ward, which persons were present in the nursing home and communication. In addition, time and place were described through sensory impressions: smells, sounds, general atmosphere (milieu) and aesthetics. The observers’ own reflections were recorded both during the field study and afterwards, representing the main material for analysis. Thus, field notes used in this study encompassed actual situations of everyday life in the nursing homes, the observers’ impressions of what happened and the initial interpretation of these situations.

After the observation period, 15 residents in all, nine women ages 75–92 and six men ages 80–90, representing all four nursing homes, were recruited for in-depth interviews. Inclusion criteria were being age 65 or older, not being cognitively impaired, and being a resident of the nursing home for 1 month or longer. The clinical nurses were asked to give the researcher a list of residents they regarded as having the ability to give consent to participate in an interview, as well as the physical and mental capacity to be interviewed. The researcher contacted the residents consecutively, handed out the cover letter and read it aloud when requested. The residents consented orally to participation, and residents who were able to write signed a written consent form. To acquire an information-rich description of the informants’ experiences, a narrative approach was used, with questions such as “*Tell me how your day is”* or “*Tell me about when you moved into the nursing home”* to encourage the informant to freely talk about his or her life in the nursing home, both positive and negative experiences. The author of this article conducted all interviews. The interviews were tape-recorded, and the interviewer also took notes that described the setting and summarized the general impression of the interview (Additional file 1).

### Analysis

The ethnographic approach require that data analysis take place in the same time frame as data collection and involve an iterative process [35]. The field notes and notes on the informal conversations with staff were first coded into meaningful entities. Then, all data were sorted into main categories, ensuring association and exclusiveness. Resident interviews were transcribed verbatim, retaining frequent repetitions, pauses, and emotional expressions [41]. The transcripts were first read through while listening to the tape recording, and a matrix of the first general themes was constructed. The analysis then moved into meaning condensation and coding. Meaningful entities in the transcripts were identified, and the text or expressions of the interviewees were sorted into more specific categories. Finally, by comparing and contrasting the content in each category, meaning categorization was achieved [41].

### Study rigour

The credibility of the study depends on both a rigorous method in data collection and analysis, and the credibility of the researcher [42]. Reflexivity is an aspect of all social research, and this reflexivity provides the basis for a reconstructed logic of inquiry and produces justified accounts of the social world [35]. Since some features of culture are not visible to those who are part of the culture, research in one’s own field is challenging. Our presuppositions may limit the achievement of full understanding of the nursing home’s culture, thus threatening the credibility of the findings [43]. Reflexivity involves a process of examining both oneself as a researcher and one’s relationship to the research context. Personal reflexivity refers to how our values, beliefs and presuppositions influence our understanding of the nursing home’s culture. For instance, during observation of nursing home practice, our own work experience from nursing homes might be used as a reference. Reflexivity encompasses to make the familiar unfamiliar by taking an outside perspective. On the other hand, our experience as nurses and familiarity with nursing home services can be used deliberately to trigger further exploration of the environment in the actual nursing home being observed, thus enhancing the richness of data acquired [44]. Contextual reflexivity involves attempts to identify the foundations of knowledge and the implications of findings of the study. For example, knowledge acquired from previous research may influence the focal point of observations; thereby determine what to lay emphasis on and what to take no notice of. To strengthen the study’s analytical rigour in the area of dependability and confirmability, the researcher and the research assistant met throughout the process to review the data, reflect on interpretations and discuss the findings [41].

### Ethical considerations

The study was approved by the Regional Committee for Medical and Health Research Ethics, Health Region Mid-Norway (ref. no 4.2008.190). During the observation study, the observers aimed at informing the staff, the residents, the residents’ next-of-kin and visitors about the study’s aim and data collection methods to clarify the observers’ roles at all times. Consent was obtained from all participants by asking both the carer and the resident for permission to observe in each situation where the researchers participated in care activities. The staff and the resident could refuse to be observed, and the residents’ families were requested to speak on behalf of those who were not competent to consent. However, in order not to disturb the daily activities and normal life in the nursing home, the researchers tried to blend in as nurses. This could have led to misunderstandings about our roles, even if we repeated the information occasionally. For the in-depth interviews, informants gave oral or written consent. There were no instances where family members gave consent on behalf of or in addition to the residents. In all reports and published material, the anonymity of individuals was ensured by avoiding identifiable characteristics in narrative descriptions.

## Results

**Fig. 2:**
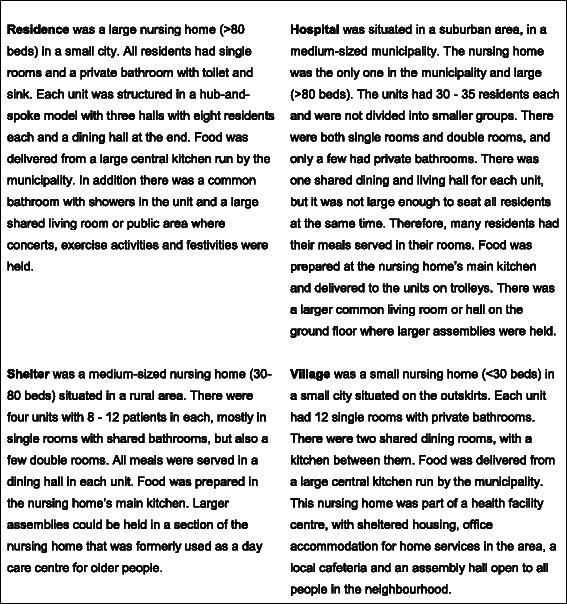
Descriptions of the four nursing homes

Figure 2 gives a description of each nursing home, focusing on how each one organized the service, ward size, architecture and food services. These descriptions were used together with the field notes, expressing similarities and differences between each nursing home.

After the initial analysis of the descriptions of the nursing homes, field notes, and researchers’ reflection notes, different or convergent patterns of regular structure and interaction in the nursing homes were found, describing the nursing home’s corporate culture. Further, an approach that described the nursing home organizations as ‘personalities’ was used. Three factors, conformity, group trait and distinctiveness, which have been used for describing the formation of human personality [45] but also organizational cultures [46], emerged, and this formed three statements for analyzing the nursing home’s ‘personality’, see Table 1. The three statements were:

‘Every nursing home is like all other nursing homes.’ (Conformity)

‘Every nursing home is like some other nursing homes.’ (Trait)

‘Every nursing home is like no other nursing home.’ (Distinctiveness)

**Table 1:** Nursing home’s corporate culture: Overview of categories and subcategories

Conformity: ‘Every nursing home is like all other nursing homes’	Trait: ‘Every nursing home is like some other nursing homes’ (typologies)	Distinctiveness: ‘Every nursing home is like no other nursing home’
Standardized basic care	Residency oriented – All residents’ rooms are similar – Group orientation – Little privacy –Institutional environment Medical oriented –Emphasize physical care – Problem orientation – Professional nursing care – Large wards Safeguard oriented – Integrated into the local society – Individualized care –Emphasis on environment similar to residents’ own homes Family oriented – Flexible routines – Residents treated as family members – Individual orientation	Unique management
Similar staffing		
24-hour service for older residents >65 years		Size of ward
Professional communication		
Standardized environment		
Similar organization of care		
		Learning organization
Common activities offered		
		Flexible organization

From the analysis of the resident interviews, two main categories of what residents viewed as important for high quality of care and considered as having met their needs and expectations emerged: ‘The nursing home as my home’ and ‘Interpersonal care quality’. See Table 2 for an overview of categories and subcategories. Further overview of the analysis process and findings from the resident interviews are provided in previously published articles by the author et al. [4, 36].

**Table 2:** Resident interviews: Overview of categories and subcategories

The nursing home as my home	Interpersonal care quality
‘Being at home in a nursing home’	‘Care for and alleviation of medical, physical and psychological needs’
‘Paying the price for 24-hour care’	‘Protecting the resident’s integrity’
‘Personal habits and institutional routines’	‘Psychosocial well-being’
‘Meaningful activities for a meaningful day’	

### Every nursing home is like all other nursing homes

This statement described how nursing home organizations developed their services according to a perception of what nursing home residents in general need and expect. In the field study, we observed that all nursing homes had organized the day in a similar way with routines for daily activities such as meals, and caring procedures such as washing. The nursing homes were also structured with similar interior designs for patient rooms, common rooms and nurses’ offices, with standard institutional furniture (see Fig. 2). The nursing homes were staffed with RNs on a 24-hour basis, which indicated the need for advanced health care among the residents. The communication in the nursing homes was characterized by professional terminology used between the staff and friendly professional nurse - patient communication. Family members or visitors were often regarded as guests and not as part of the nursing home community. Extracts from the field notes illustrate conformity aspects of the nursing homes:

*A typical Norwegian nursing home, spacious common areas made as homelike as possible. Bright colours on the walls and floor. White curtains and large ocean-view windows. Numerous plants and flowers. Solid chairs for everyone and plenty of space for people who rely on a walker. The whole ward is nicely decorated. The dementia unit has been furnished with cutlery, furniture, pictures, curtains and other objects that one would have expected in a private home. A pleasant-looking ward that enables both the staff and residents to thrive and identify as their own place. The staff room is easily accessible, which certainly has a practical purpose for the personnel, patients and family member. The basic care seems to be the same as what patients normally receive in a nursing home. (From Shelter, recorded by research assistant)*

*Each floor is divided into three units, of which one is the sheltered unit. There are 12, 9 and 8 residents, respectively, in each of the units. On the day shifts the units are attended by three, two and two staff members, respectively, all of whom are licensed practical nurses, assisting nurses or sometimes registered nurses. On the evening shifts there are two, two and one workers, respectively, and there are two on night shifts on each floor. There are two registered nurses on the day shift, one on the evening shift and one on call during the night. (From Shelter, recorded by researcher/author)*

*The staff members discuss how they divide the work between themselves or consult each other whenever they are uncertain about something. The discussions take place in the staff room, corridors or in the living room. Mostly, the conversations concern which resident has been attended to, how their needs should be addressed, observations or division of labour. The staff members talk to the patients and the patients respond as far as they are able to. A number of them depend on the staff to initiate communication. (From Village, recorded by researcher/author)*

This illustrated the conformity of nursing homes in general. The nursing homes had common structures such as staffing, similar organization of nursing home services and a standardized environment (see also Table 1). Nursing home culture in all four nursing homes included common norms for basic care and professional communication between staff and between staff and residents.

These typical characteristics of nursing homes in general were in line with some of the common needs of the residents. The residents stated that routines and a certain rhythm of the day were important to their feeling safe and taken care of. One of the interviewed residents in Hospital said: ‘I feel well here. I feel safe. I get food in the morning and get help to be dressed up’. On the other hand, there were fewer opportunities to maintain their personal habits if they did not correspond to the institutions routines. The conformity of the nursing homes was regarded as quite rigid with little or no possibility to change or participate in development. The following citation is from one of the resident interviews at Residence:

*Interviewer: If there is something that you would prefer to work differently – would you know how to do this? Is it possible for you to make any changes to things?*

*Resident: No. No, I’m not able to – I haven’t thought about this. I don’t think so. I think it’s not possible to do this. I can’t think of any ways to make things work differently.*

Thus, for some residents, the activities offered in the nursing homes suited them fine, but for others or at other times, there were no activities that were meaningful for them. This was expressed by a resident at Shelter who said: ‘No, no, I do not participate in those [activities]’.

### Every nursing home is like some other nursing homes

This statement expressed organizational and cultural types in the sense of organizational or cultural assumptions being present in some of the nursing homes but not in all. The nursing home type represented a corporate culture among staff at all levels and in incorporated visions or statements describing the nursing home. The four nursing homes were different in some aspects, but at the same time had characteristics that are common for other nursing homes that we and some of the staff had experienced previously. These characteristics formed four typologies: ‘residency oriented’, ‘medical oriented’, ‘safeguard oriented’ or ‘family oriented’. The focus of care was present throughout the organization, in the daily activities, communication, artefacts, espoused values and underlying assumptions, as interpreted by the observers. Four field notes from the different nursing homes expressed this:

*Residency oriented (Residence):*

*The patient room has one bed, a bedside table, a private table and a TV. On the wall a calendar has been fastened with a thumb tack. Apart from this there are not pictures on the walls. The man has been living here for 7 months. The bathroom, which is extremely small, smells of contaminated urine. Clean diapers, towels, gloves, pads, catheters and other things lie on a shelf. I get an impression of loneliness among the patients, as they all were seated alone. During informal conversation with one patient he states: ‘I don’t enjoy staying here, but I do it out of consideration for my wife’. The institution-like atmosphere at the nursing home feels depressing and stifling. I thought it was like living in a hotel, with very little privacy. Most people find it attractive to stay in a hotel for a while, but after a while you miss your home. There is no mention of the fact that one will not return home after completing one’s stay or ‘holiday’. (Recorded by the researcher/author)*

*One patient who is being looked after says: ‘It’s like a kindergarten’ and: ‘I do as you tell me’ (informal conversation). The patients are served meals where they sit and do not have regular places at the table. (Recorded by the research assistant)*

The definition of the residency oriented nursing home found in the present study was a main focus on providing housing and custodial care. This type of nursing home was characterized by similar rooms, little privacy and meals served in an institutional manner. Residents experienced the nursing home as a home important for quality of care, but this was difficult to achieve in the residency oriented nursing home.

*Medical oriented (Hospital):*

*In the stairs leading up to the ward there is a glass cupboard with old medical equipment. One gets the feeling of being in a hospital. The combination of homely antiques and medical equipment leaves an ambiguous impression. It is hard to tell if the nursing home wish to promote an image of a health institution or a home for the elderly. The staff members seem quite focused on routines and are anxious to complete their tasks. Still, I get a feeling that they are concerned about the individual needs of the patients and that they show a high level of compassion. The staff meetings emphasize physical and psychosocial problems, such as pain, difficult breathing, defecation, problems in getting up from bed, not feeling well, the amount of food eaten, anxiety. Social conditions are given little attention. There is no mention of whether the patients talk to each other, where they spend their time or with whom they socialize. (Recorded by the researcher/author)*

*I get the impression that a number of the staff members are competent professionals, but that the system, physical environment and organization restrict creativity and the possibility of offering more intimate, individual-oriented care. After spending a week here I have not managed to get a complete overview of the ward. (Recorded by the research assistant)*

From two of the in-depth interviews with residents:

*Interviewer: Do you feel lonely here?*

*Resident: No, I wouldn’t go that far. Still, at times, when nobody comes by to have a chat or something – it may become lonely.*

*Interviewer: What would happen if you get poorer health?*

*Resident: ‘I get bedbound; I guess’*.

The findings defined the medical oriented type of nursing home as organized mainly with a focus on the residents’ medical problems and needs. The care seemed to be professional at an individual level. However, some of the residents had doubts about the nursing home’s ability to provide adequate help if their functions declined. This satisfied the residents’ experiences of medical care quality but was an obstacle to the feeling of ‘at-homeness’.

*Safeguard oriented (Shelter):*

*The nursing home is located in a small municipality. This is a small community where everyone knows each other, and for this reason the staff members may find it easier to communicate with the residents within topics of interest for the patients. A number of patients are able to groom on their own, and only need assistance for things like combing their hair and tidying their rooms. The staff members contribute to create a relaxing atmosphere in spite of disturbing behaviour in one of the patients. The staff members discuss everyday matters with the residents, like children, family members and places where they used to live. There are also discussions about things that bother the patients, like pain, disease and poor walking function. The staff seem to know the patients well and the conversations run smoothly. The doctor is available at the nursing home 12 h a week while a junior doctor is present 7 h a week. At the doctor’s visit, the group leaders (certified nurse assistants) meet the doctors and keep track of the patients who need medical attention or matters linked to medication or similar things. The doctor wears a white doctor’s coat when attending to the patients. (Recorded by the researcher/author)*

*The nurses are conscious not to exaggerate their assistance and want the patients to carry out their daily tasks as far as possible. (Recorded by the research assistant)*

*‘I have an alarm, a string I pull… they (staff) come running [to help]’ (quote from a resident in the in-depth interview)*

Integration into the local society, individual care and a focus on a home-like environment defined the safeguard oriented nursing home. This aligned with what residents experienced as important for quality of care, both the nursing home as a home and interpersonal quality. With access to help at short notice, the residents felt safe in the nursing home.

*Family oriented (Village):*

*All residents have single rooms with bathrooms. The rooms were located on either side of the living room/kitchen with a small corridor in the middle. The staff room is not located in the central area. There is an outdoor view from all rooms. During one of our days at the ward the staff members serve cake in the living room. On another day the activity coordinators organize a party for the residents in the assembly hall. The tables are laid, cakes are served and a pensioner’s band play dance music. It looks like an enjoyable activity in which many of the residents take part. A number of the residents are on first name terms with the staff members and call out their names whenever they need assistance. The employees eat their lunch in the living room. Three residents who happen to be in the living room have a conversation with the staff members. It is a comfortable setting with sounds and smells that we associate with an ordinary home. The staff members claim that they have become a closely knit group as they have worked together for many years. During this time they have seen many directors come and go. They feel they can run the affairs on their own. (Recorded by the researcher/author)*

*The ward is modern, cosily furnished and with attractive colours. The small number of patients makes it a comfortable place to be. I get the impression that the staff are doing a good job and that their relations to the patients and family members are cordial. A nurse states that the ward becomes like a family and that emotional bonds develop between the staff and patients. (Recorded by the research assistant)*

*‘I feel that they are fond of me, they often give me a hug’ (quote from a resident in the in-depth interview)*

The family oriented nursing home was, in this study, defined as being flexible according to each resident’s needs, with an individual orientation. The professional care was associated with family-style care. The residents emphasized that aspects of ‘at-homeness’ and interpersonal quality could be fulfilled in this type of nursing home.

### Every nursing home is like no other nursing home

The distinctness or uniqueness of each nursing home’s ‘personality’ was expressed in this statement. Certain patterns of structure, cultural assumptions and interactions in the nursing homes were observed to be different in each nursing home and formed a distinction among them. This distinctiveness emerged as individual organizational and corporate cultural features of each nursing home, based on historical or environmental adaptation over time. This observation note was made:

*The absence of visible management caused that those who had worked at the ward for some time had gained a certain informal power, enabling their views and attitudes to influence the activities. There was not a lack of official routines or planning tools at the ward, though the absence of control and management made it necessary for each staff member to identify the relevant regulations, and the implementation of these became the responsibility of the individual service-provider (From Residence, recorded by the researcher/author)*

The uniqueness of the nursing home seemed to fluctuate depending on the stability of staff or residents. For instance, the physical plant for Village had been relocated several times, but most of the staff and residents remained the same. The staff felt they were like a family, with the same level of commitment to each other even if they had moved into a new building and new place. The appointment of a new nurse disturbed the environment, especially in the way communication between the staff and residents was accomplished. This could be illustrated by one of the field notes from this nursing home:

*I talked with many of the nursing assistants who seem quite reflective, and they are concerned about providing good services to the residents. It seems like they are not satisfied with the nurse [refers to the new nurse], which makes it a bit difficult to be present there. (From Village, recorded by the research assistant)*

The residents in Village had complex needs, and many of them were referred to this nursing home because of their special needs for individual care. The residents who experienced a high quality of care in Village with these unique features had lived there for a long time, or their special needs could be met in such a nursing home, as one resident explained in the in-depth interview:

*Many of the nursing home residents are like me. We are really comfortable. I couldn’t have been in a better place. The environment was… it was a different attitude, you see, when I arrived here. I felt that it was something entirely [different]. But as I said, I wash on my own. I don’t require much, but when I need something they always make themselves available. I feel they really care for me.*

Quality of care seemed to be dependent on whether the nursing home could adapt and form uniqueness in the organization that closed the gap between the services offered and the needs and expectations of the residents. For instance, in Shelter many of the residents had fewer medical needs but had moved to the nursing home because they could not stay at home due to lack of home health services. This nursing home had organized small groups of residents led by certified nursing assistants (CNAs) who had worked there a long time and knew the residents well. Likewise, one resident with extensive medical needs living in Hospital felt that ‘the service was as good as it could be’ (quote from in-depth interview with the resident), even though his feeling of ‘at-homeness’ was not present. Village had organized the care with primary nurses or contact persons (CNAs) who were responsible for each resident’s total care-physical, psychological, social and spiritual. The primary nurse developed care plans and normally cared for his or her resident almost like a family member. It seemed to be a good system for individually adjusted care. However, new care staff who were still not aware of the norms in the culture threatened the understanding of what was important for quality of care from the staff’s point of view and as experienced by residents.

## Discussion

This ethnographic study showed that the nursing homes had relatively stable corporate cultures described as ‘personalities’ with some characteristics that were common to all nursing homes (conformity) and typical traits that were present in some nursing homes forming four typologies; residency oriented, medical oriented, safeguard oriented or family oriented. Further, the nursing homes had developed some ‘personality’ characteristics that were like no other nursing home (distinctiveness). Nursing home residents experienced ‘at-homeness’ (‘the nursing home as a home’) and ‘interpersonal important for quality of care.

Part of each nursing home’s ‘personality’ encompassed conformity expressed as ‘Every nursing home is like all other nursing homes’. Even though residents accepted standardized care and some residents were comfortable with common routines in the nursing homes, it is necessary to point out that residents should not be responsible for creating a healthy environment for themselves by adapting to existing organizational culture. Designing nursing homes as conforming organizations might be the reason why, in the same nursing home, residents perceived the day as busy or boring, meaningful or devastating. Standardized care illuminated a corporate culture where nursing home residents are seen as merely subject to the culture rather than part of the nursing home culture. Institutional rules, procedures, and environment, and a high degree of conformity to corporate culture can be obstacles to achieving quality of care [4, 31, 32]. However, an area for discussion could be to what degree the ‘personality’ can be changed to close the gap between nursing home corporate culture and residents’ perception of what is important for quality of care. A study found that residents are customized to organizational practices and feel they have little possibility of challenging these practices [47]. In addition, it is important to recognize the basic human right to be treated equally, although, in some instances this means treating residents differently based on differences in their needs and preferences. The distinction between ‘equality’ and ‘sameness’ is important to recognize in nursing home organizations to prevent a service that provides only standardized care, regarding this as the most fair and valued health care service [48].

The statement ‘Every nursing home is like some other nursing homes’ highlights a part of each nursing home’s trait, and this created four specific typologies. To some extent, each specific trait of the nursing homes’ corporate culture seemed to be in line with their residents’ experience of quality of care, whether it was ‘the nursing home as my home’ or ‘interpersonal quality’. Quality of care experiences from the residents’ perspectives required an assurance that their priorities could be met and that the interpersonal interactions corresponded to their values. However, holding onto a specific nursing home corporate culture based on the historical composition of residents might be obstacles to organizational development. Being open to change when the characteristics of the resident group changes is important to develop trait characteristics that align with residents’ needs and expectations. As presented in this article, the nursing home community or a nursing home’s ‘personality’ is formed by all stakeholders including the residents, their relatives and the staff, and structures such as the physical environment. This means that it is important to foster a balanced relationship among all parties. Respect for the residents as individuals with different needs is the essential attribute in a personalized model of care [13, 17, 49, 50]. Relationship-centred care is suggested to enhance the development of a shared understanding of all residents’, staff’s and family members’ needs and values, and a feeling of all being included as members of the nursing home community [51–53]. Being recognized as an individual is a crucial aspect of life in a nursing home, contributing to meaningfulness in which one’s humanity is preserved [14, 54]. Quality improvement actions in nursing homes should be based on an approach where individual needs and expectations are assessed and care is individually adjusted [23, 55].

In the in-depth interviews residents underpinned the importance of seeing each resident as an individual person. Observations made in the field study confirmed that quality of care was dependent on the nursing home’s uniqueness, expressed in the statement ‘Every nursing home is like no other nursing home’. This was in line with the current residents’ values. The findings in the present study showed that a corporate culture that emphasized safety or creating a family-type nursing home could foster such values. Nursing homes with fewer residents in each ward or organizing the nursing home into smaller groups could facilitate a closer relationship between staff and residents. However, changes in the staff such as employing new care staff led to difficulties accepting alternative practices. New employees might not be familiar with the specific norms and values in the corporate culture. The differences between the formation of the ‘personality’ in Residence, Hospital, Shelter and Village respectively, also point out that nursing homes as organizations consist of clinical units that can be viewed as clinical microsystems [56]. The idea of clinical microsystems is that they are the basic building blocks of interaction where care is provided and quality is achieved or not. A corporate culture that supports quality of care is where each team member’s individual and complementary skills and abilities are used together, supporting a well-functioning microsystem [57, 58]. However, a key question is whether corporate culture as an internal variable can be manipulated to influence the nursing home’s performance or outcomes for its residents [28]. Believing that change in an organization’s culture is achieved by taking control of staff members’ behaviours tends to be overly optimistic, partly because there are likely to be multiple subcultures and countercultures competing to define the nature of situations [28]. Culture change is a continuous learning process, not a one-time event [59]. For change to happen, all staff members and the management must recognize the organization’s own problems and must share the values of a new culture [12, 33, 60]. If all employees understand the reasons for change and decision-making is moved to the clinical microsystem, it is more likely that changes in behaviours leading to better outcomes for the residents will occur [16, 61, 62].

Stable and sometimes rigid corporate cultures were, in some instances, obstacles to delivering nursing home services that corresponded to the residents’ experiences of what was important for quality of care. However, the corporate culture of nursing homes with unique distinctiveness that had adjusted to their present nursing home residents’ needs and expectations seemed to have succeeded most in terms of quality of care. Nursing homes are a complex phenomenon where both supportive care and curative services compete for the time and energy of the staff. At the same time, adjusting the health service in nursing homes to suit both the individual resident and the organization is challenged by varying and often progressively complex needs of the residents [4, 63–65]. Being a long-term resident in a nursing home implies a focus on privacy, the living place and space, as well as the availability of stable caregivers safeguarding the health service [66].

The strength of the present study is the rigorously methodological approach aiming at covering both the staff’s and residents’ perspectives. However, the significance of management effects was beyond the scope of this study, and this should be explored further. A limitation of the study might be that only four nursing homes were observed during a relatively short period of time. Variations and organizational events that occur infrequently could therefore have been missed. On the other hand, in informal conversations with staff, this was highlighted, leading to a better understanding on the part of the observers about what happened was random or part of the nursing home corporate culture. The researchers’ subjective observations and interpretations may have caused bias in the findings. However, we were aware of this problem and tried to reflect upon this to enhance a fair description of the nursing homes and the expressions of the residents [67]. Because the observers are nurses, and since the present research was conducted in a cultural context that we had experienced as professionals in other settings, the observations and field notes may have been influenced by our professional knowledge. Thus, significant observations could have been missed as parts of everyday life in the nursing homes were taken for granted. The researcher and research assistant discussed this concern both during the observations and at the end of the observation period in each nursing home to uncover such presumptions. Extended field notes and observers’ impressions are provided in the article to make it possible for readers to align the findings to their own practice [35].

## Conclusion

Population projections and predicting the needs of residents in nursing homes in a 10 to 20 years perspective is a complex task, and is associated with unanticipated factors. We know the most about the demographic situation in the future but less about the needs, expectations and preferences of older people in 2030. This study provides a greater understanding of organizational and cultural factors that influence residents’ perceived quality of care in nursing homes. It is crucial that the organizations are flexible and willing to prepare for cultural changes in order to close the gap between the nursing home’s corporate culture and residents’ experience of quality of care as ‘the nursing home as my home’ and ‘interpersonal quality’. Moreover, it is important that health organizations learn from their clinical practice and that rigid ‘personalities’ consisting of conforming structures, traits and distinctions found in the present study are prevented from becoming cemented in the organization. A sustainable nursing home service needs to be characterized by learning, openness to change and actually implementing practices that respond to the resident and his or her family’s values.

## Additional file

Additional file 1: **Interview guide for the interviews of residents in nursing homes.** (DOC 28 kb)

## Abbreviations

RN: Registered nurse
CNA: Certified nurse assistant
