# Supplementary material for: Understanding organizational and cultural premises for quality of care in nursing homes: an ethnographic study
Source: BMC Health Serv Res. 2015 Nov 13;15:508. doi: 10.1186/s12913-015-1171-y (PMC4643525; doi:10.1186/s12913-015-1171-y)
Supplement: Additional file 1: — Interview guide for the interviews of residents in nursing homes. (DOC 28 kb) [file 12913_2015_1171_MOESM1_ESM.doc]

**Interview guide for the interviews of residents in nursing homes:**

**Introduction to the interview:** I would like to interview you about what you feel is important for you when it comes to the quality of the nursing care, how you like it here and how your daily life is here in the nursing home.

**Theme 1: The physical environment in the nursing home**

Tell me what your day looks like. What do you do during a day? What do you think about your private room? The living room and dining room? Who decided which room you got and how to furnish it?

**Theme 2: Psychological needs, well-being and thriving (Quality of life)**

If you should describe a good day, what does it contain? How are you feeling when you experience a good day? How can others understand that you are comfortable? Are there things you miss? What do you do if you want to have some changes to your service?

**Theme 3: Social needs, relationships (nurses or other residents)**

Do you feel that the staff knows you and your needs? In what way does the staff show that they care about you and your interests? Is it easy for you to understand and hear what the nurses and the physician say? Can you give me examples of situations when you felt you had a special contact or relation to staff members/nurses? Who of the other residents do you talk with? What do you talk about?

**Theme 4: Nursing care and treatment**

What do you get help to perform when it comes to personal hygiene, grooming, dressing and toileting? What do you think about the food and meals? Do you have problems with eating or appetite? What is done to meet your needs? Why did you move to the nursing home? How do you feel your health is right now? What kind of medical problems do you have, and what is done to help you?

**Theme 5: Human dignity, human rights, respect and self-determination**

Can you describe a situation when you felt very well cared for? What does it mean that you feel well cared for? Are there some particular persons or nurses in the staff that take care of you in a better way? How can you notice that these persons care more for you? Who or what do you feel decides over your day? To what degree do you feel you can decide things for your self? How dependent or independent do you feel?
